# Substrate-mediated strain effect on the role of thermal heating and electric field on metal-insulator transition in vanadium dioxide nanobeams

**DOI:** 10.1038/srep10861

**Published:** 2015-06-04

**Authors:** Min-Woo Kim, Wan-Gil Jung, Hyun-Cho, Tae-Sung Bae, Sung-Jin Chang, Ja-Soon Jang, Woong-Ki Hong, Bong-Joong Kim

**Affiliations:** 1 School of Materials Science and Engineering, Gwangju Institute of Science and Technology, Gwangju 500-712, South Korea; 2 Jeonju Center, Korea Basic Science Institute, Jeonju, Jeollabuk-do 561-180, South Korea; 3 Department of Chemistry, Chung-Ang University, 84 Heukseok-ro, Dongjak-gu, Seoul 156-756, South Korea; 4 School of Electrical Engineering and Computer Science, Department of Electronics, Yeungnam University, Gyeongsangbuk-do 712-749, South Korea

## Abstract

Single-crystalline vanadium dioxide (VO_2_) nanostructures have recently attracted great attention because of their single domain metal-insulator transition (MIT) nature that differs from a bulk sample. The VO_2_ nanostructures can also provide new opportunities to explore, understand, and ultimately engineer MIT properties for applications of novel functional devices. Importantly, the MIT properties of the VO_2_ nanostructures are significantly affected by stoichiometry, doping, size effect, defects, and in particular, strain. Here, we report the effect of substrate-mediated strain on the correlative role of thermal heating and electric field on the MIT in the VO_2_ nanobeams by altering the strength of the substrate attachment. Our study may provide helpful information on controlling the properties of VO_2_ nanobeam for the device applications by changing temperature and voltage with a properly engineered strain.

Strongly correlated materials (SCMs) exhibit a variety of remarkable physical properties, such as metal-insulator transition, high temperature superconductivity, and colossal magnetoresistance, resulting from complex interplays between electrons, phonons, spin, and lattice degrees of freedom^1^,^2^. Among the widely studied SCMs, vanadium dioxide (VO_2_) has been highly recognized due to its first-order metal-insulator transition (MIT), coupled with a structural phase transition from a high-temperature tetragonal rutile-type phase (R, space group *P4*_*2*_*/mnm*) to a low-temperature Monoclinic phase (M, space group *P2*_*1*_*/c*) at the temperature of ~340 K^3^,^4^.

In spite of the intense controversy over the mechanisms of the MIT in VO_2_, it has been generally believed that the transition is driven by either strong electron-electron interaction (Mott transition),^5^,^6^ electron-phonon interaction (Peierls transition)^7^, or a combination of both mechanisms^5^,^8^,^9^. Moreover, the properties of the MIT in VO_2_ are significantly affected by stoichiometry^10^,^11^,^12^, doping^13^,^14^,^15^, size effect^16^, external strain^17^,^18^, interfacial strain^4^,^19^,^20^, and defects^21^,^22^. To effectively investigate those issues, such single crystal nanostructures have been widely exploited, where the beam width is comparable to the domain size. The single domain nanobeam takes an advantage of fabricating potential applications in functional devices such as phase-change memories^23^, bolometric sensors^24^, and stationary Hadamard shutters^25^, which benefit from a much sharper change in electrical and optical properties.

In particular, the strain imposed on the VO_2_ nanobeams (or nanowires) have been considered an important topic both scientifically and technologically. For examples, the strain involved in the VO_2_ nanobeams can be manipulated to tune the transition temperature of up to ±50 K by loading tensile or compressive external uniaxial strain in the VO_2_ nanobeams^26^, which are often employed when transferring the nanobeams onto flexible large scale substrates^27^. The single domain nanobeam provides a fascinating model system to study the electrical switching that accompanies structural domain formation. It was demonstrated^14^ that the substrate-induced strain leads to the spontaneous formation of alternating metal-insulator domain patterns along the nanobeam axis, coincided with step-like changes in resistance with temperature around the MIT. Moreover, they engineered the transition temperature by mechanically bending the nanobeam^28^. Recently, a research group measured electrical conduction of single crystal VO_2_ microbeams across the MIT at various strain and temperatures, and observed a universal resistivity for the insulating phase near the MIT^29^. To date, however, as compared with previous reports^1^,^4^,^19^,^28^,^30^,^31^,^32^,^33^,^34^,^35^,^36^, the conduction mechanisms of the MIT in VO_2_ nanobeams have not been clarified when both thermal heating and electric field are applied to a single VO_2_ nanobeam with different strength of substrate attachment.

Herein, we have investigated the effect of substrate-mediated strain on the role of thermal heating and electric field on the metal-insulator transitions in two terminal VO_2_ nanobeam devices in which the VO_2_ nanobeams are placed on the substrates *via* solution-dropping and polydimethylsiloxane (PDMS)-transferring methods. The electrical measurements with varying temperature and voltage yield hysteresis, and their sizes are relatively larger in PDMS-transferred nanobeam devices because the insulator-to-metal transition occurred at higher temperature and voltage. By evaluating the threshold voltages (V_TH_), we found that the insulator-to-metal transition and the metal-to-insulator transition are controlled by a collective motion of carriers and a joule heating effect in the nanobeams, respectively, giving a trend that the V_TH_ decreases with increasing T. Notably, for the PDMS-transferred VO_2_ nanobeam devices, the V_TH_ for the insulator-to-metal transition is not compensated by heating at high T, representing a significant level of electric field is mandatory.

## Experimental

All of the VO_2_ nanobeams used in this work were grown by a vapor phase transport process, as described in elsewhere^37^. We find that the VO_2_ nanobeams are single-crystalline with a monoclinic structure (Supplementary Figures S1). For these measurements, we used FEI transmission electron microscopy (TEM) Tecnai operated at 300 KeV. The average diameter and length of VO_2_ nanobeams used in this study were found to be approximately 110.5 nm and 4.1 μm, respectively, from field emission scanning electron microscopy (FESEM) images of roughly 60 different nanobeams that we measured statistically (Supplementary Figure S2).

**Figure 1:**
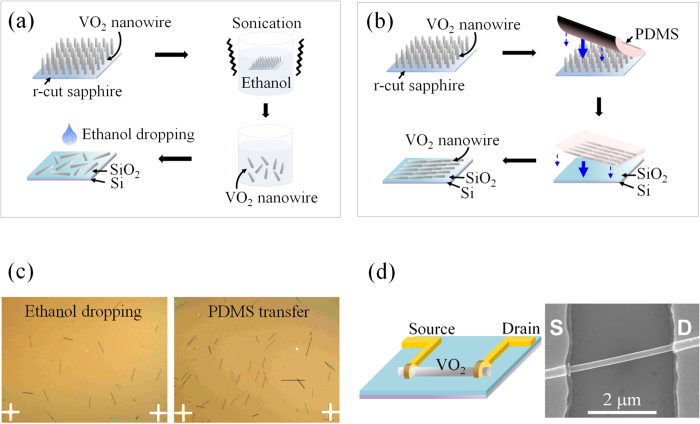
Schematic illustration showing two different types of transferring processes of VO_2_ nanobeams onto the SiO_2_/Si substrate. Schematic illustration depicting a solution-dropping method (**a**) and a PDMS- transferring method (**b**). (**c**) Optical images of VO_2_ nanobeams transferred on the SiO_2_/Si substrate. (**d**) A schematic diagram (left) and a SEM image (right) of the two-terminal devices fabricated using VO_2_ nanobeams.

To investigate the combined effects of thermal heating and electrical field on the MIT in VO_2_ nanobeams, we fabricated two different types of two-terminal VO_2_ devices with the nanobeams placed on the substrates through solution-dropping and polydimethylsiloxane (PDMS)-transferring methods. For the two-terminal VO_2_ nanobeam devices, metal electrodes consisting of Ti (80 nm thick)/Au (100 nm thick) were deposited by an electron beam evaporator, and they were defined as sources and drains by photolithography followed by lift-off processes. The distance between the source and drain electrodes was about 3 μm, as shown in the inset of Fig. 1d. For the electrical measurements of the two-terminal devices using single-crystalline VO_2_ nanobeams, a total of 9 devices were fabricated and characterized - six and three devices by solution-dropping and PDMS-transferring methods, respectively. The electrical properties of the nanobeam devices were measured using a semiconductor characterization system (Keithley 4200-SCS).

We carried out simulations by modeling Joule heating in the nanobeam using a COMSOL Multiphysics software. Here, we assumed a constant electrical conductivity at the temperature range showing completely insulating phases for the VO_2_ nanobeams.

## Results and Discussion

Figures 1a,b show schematic illustrations of the transfer-processes of VO_2_ nanobeams onto the silicon wafer on which a 100 nm-thick SiO_2_ layer is formed by solution-dropping and PDMS-transferring methods, respectively. For the solution-dropping method, as-grown VO_2_ nanobeams on a r-cut sapphire were released by sonication in ethanol and the nanobeam-dispersed solution was dropped on the SiO_2_/Si substrate (Fig. 1a), whereas for the PDMS-transferring method, a PDMS stamp was attached and pressed onto the VO_2_ nanobeams grown on the r-cut sapphire substrate, followed by the detachment of the PDMS slab from the substrate. Then, the PDMS with adhered VO_2_ nanobeams was strongly pressed against the transfer substrate (SiO_2_/Si substrate) at a moderate temperature to make a firm contact between the transfer substrate and the PDMS stamp during mechanical transfer. The PDMS then was peeled off from the substrate, resulting in the transfer of VO_2_ nanobeams from the r-cut sapphire onto the SiO_2_/Si substrate (Fig. 1b). Figure 1c shows the optical microscopy images of the VO_2_ nanobeams transferred to the SiO_2_/Si substrates by the solution-dropping and PDMS-transferring methods. Figure 1d shows a schematic illustration and a representative scanning electron microscopy (SEM) image of a two-terminal device fabricated using a VO_2_ nanobeam which was transferred by the two different methods.

**Figure 2:**
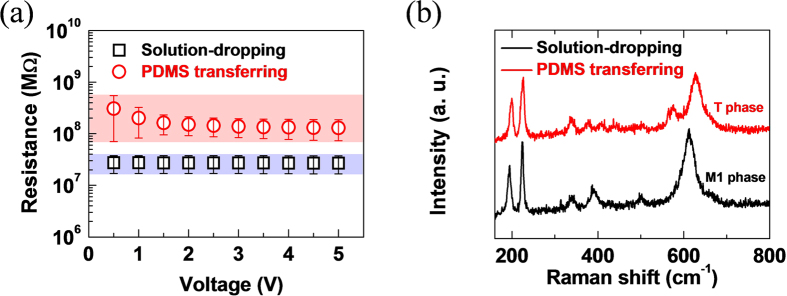
Room temperature resistance of a VO_2_ nanobeam and its Raman characteristics. (**a**) Resistance as a function of the applied voltage at room temperature for the solution-dropped and PDMS-transferred VO_2_ nanobeam devices. (**b**) Raman spectra for the solution-dropped and PDMS-transferred nanobeams, respectively.

Hu *et al.*^31^ demonstrated that the resistance of the VO_2_ nanobeam devices increased in the response to an increase of the tensile strain in the nanobeam, whereas it decreased with a compressive strain. Similarly, in our work, the resistance versus the bias voltage plot at room temperature (Fig. 2a), where both nanobeams are insulators, provides a substantial evidence that the PDMS-transferred nanobeam devices are tensile-strained in contrast to the solution-dropped nanobeam devices. Notably, although there is the inevitable device-to device variation, the current levels of the solution-dropped VO_2_ nanobeam devices are relatively higher than those of the PDMS-transferred VO_2_ nanobeam device (Supplementary Figure S3). This is also well supported by the Raman scattering characteristics of the nanobeams prepared by the two methods, as shown in Fig. 2b. From this, the PDMS-transferred nanobeam is the triclinic T phase^18^,^38^,^39^, compared to the monoclinic M1 phase of the solution-dropped nanobeam. The existence of the T phase in the PDMS-transferred VO_2_ nanobeams can be attributed to two possible reasons: (1) the influence of composition (e.g. excess oxygen or doping non-uniformity) and (2) the possibility of the tensile strain induced by mechanical transfer using a PDMS stamp^18^,^33^,^38^,^40^. The T phase is an intermediate transitional phase between M1 and M2 phases^4^,^18^,^38^,^39^. In the M1 phase (M1, *P*2_1_/*c*), the vanadium atoms are paired and tilted, forming zigzag chains along the c-axis in the rutile R phase (c_R_)^4^,^18^,^38^,^39^. In contrast, the M2 phase monoclinic phase (M2, *C*2/*m*) has two types of V chains consisting of equal-spaced tilted V chains and paired V chains, this phase has only the vanadium atoms in one sub-lattice remaining as zigzag chains, while the other half of the vanadium atoms is strongly dimerized along the c_R_^4^,^18^,^38^,^39^. In our previous report^38^, we especially demonstrated that the T phase is not due to the contribution of excess oxygen or metal–ion dopants using the energy-dispersive X-ray spectroscopy (EDS) studies and the Raman scattering. Accordingly, although the tensile strain can be induced by the influence of composition (e.g. excess oxygen), the tensile strain in our sample can be introduced by strongly adhesive interactions between the nanobeam and the substrate, resulting from the mechanical transfer using the PDMS stamp^38^,^39^.

**Figure 3:**
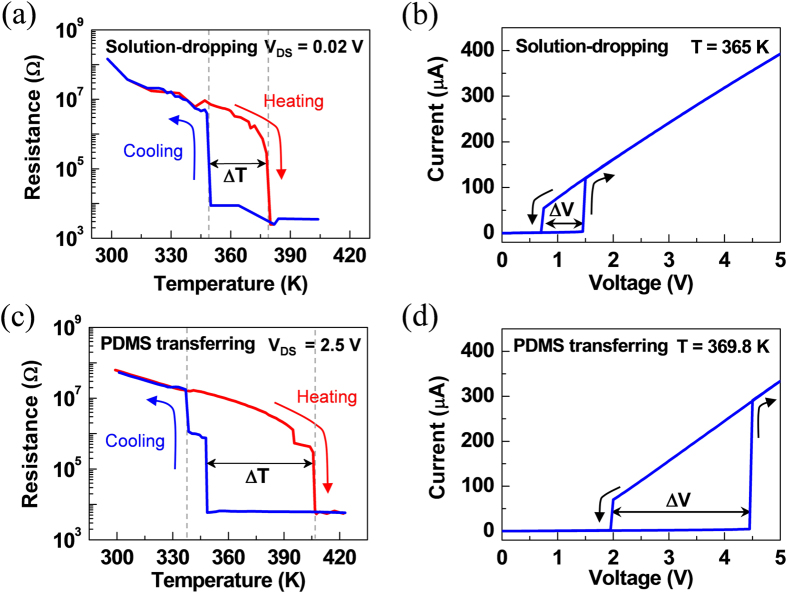
Temperature-dependent resistance for a VO_2_ nanobeam. Representative temperature dependence of the resistance during heating and cooling cycles for the nanobeam transferred by (**a**) a solution-dropping method and (**c**) a PDMS-transferring method. The solution-dropped and PDMS-transferred nanobeam devices were measured at V_DS_ = 0.02 and 2.5 V, respectively. Representative current (I)-voltage (V) characteristics measured consecutively by varying the applied voltage both in the forward- and reverse-sweep from 0 to 5 V (**b**) for the nanobeam transferred by a solution-dropping method and (**d**) for the nanobeam transferred by a PDMS-transferring method. The two-terminal VO_2_ devices fabricated using the solution-dropping and PDMS-transferring methods were measured at 365 K (Fig. 3b) and 369.8 K (Fig. 3d), respectively.

In order to understand the influence of the strain building-up during the nanobeam-transfer on the phase transition of the VO_2_ nanobeams, we examined the electronic transport properties of the two-terminal devices connected in series with an external resistor (10 kΩ) to limit the current flow during the phase transition of the nanobeam. Figures 3a,c show the representative electrical resistance as a function of temperature for the solution-dropped and PDMS-transferred nanobeams, respectively. For the solution-dropped nanobeam device, the electrical resistance starts from the insulating state with a gradual decrease as temperature increases, and then it drops abruptly by about 2.5 orders of magnitude at the insulator-to-metal transition temperature of about 375 K under a constant voltage (V_DS_) of 0.02 V. This transition temperature is much higher than the nanobeams without electrodes, which experience the transition at approximately 340 K^3^. This behavior of resistance is reversed as temperature decreases. However, the metal-to-insulator transition occurs at about 347 K – similar to the non-contacted nanobeams – at which the resistance rapidly increases by about three orders of magnitude. Then the resistance slowly increases with the similar rate during the heating cycle. This temperature dependence of resistance of the solution-dropped nanobeam device clearly presents a hysteresis at the phase transition temperature when heating and cooling are employed, with the hysteresis width (ΔT_MIT_) of approximately 30 K. The presence of thermal hysteretic behavior of VO_2_ nanobeam devices can be associated with the first-order transition in which the phase transitions between metallic rutile and insulating monoclinic phases are significantly affected by the metal electrodes which grab the nanobeam at both ends and the interfacial strain between the nanobeam and the substrate^19^,^41^,^42^.

Compared to the solution-dropped nanobeam device, the PDMS-transferred nanobeam device exhibits distinct MIT characteristics. Unlike the solution-dropped sample, the sharp change in electrical resistance for the PDMS-transferred nanobeam device was not observed at the same applied voltage (V_DS_ = 0.02 V) (Supplementary Figures S4 and S5). It should be noted that we examined the electrical resistance as a function of temperature by applying different constant voltages for these two types of VO_2_ nanobeams to investigate the abrupt transition behavior for the PDMS-transferred VO_2_ nanobeam device in comparison with that for the solution-dropped nanobeam device. Firstly, while supplying a constant voltage (V_DS_) of 2.5 V, the insulator-to-metal transition occurs abruptly at about 405 K which is higher than the solution-dropped nanobeam device, whereas the metal-to-insulator transition takes place rapidly at the similar temperature as the solution-dropped nanobeams (~347 K). Thus the PDMS-transferred nanobeam device creates a larger hysteresis width (ΔT_MIT_) than the solution-dropped nanobeam device. We find that this trend is consistent based on the statistical results of several of the two types of devices, as shown in Table 1 (also see Supplementary Figure S6). These results indicate that the phase transition behavior in VO_2_ is strongly affected by stress/strain states, which is consistent with previous reports^17^,^43^,^44^,^45^. In Fig. 3, the PDMS-transferred VO_2_ nanobeams show quite different temperature-dependent behavior and current-voltage characteristics compared to the solution-dropping nanobeams. In this sense, we suspect that although thermal hysteresis is typically due to the first-order nature of the phase transition, the extended hysteresis width can be affected by the tensile strain across the nanobeams caused by the PDMS-transferring method^17^,^21^,^38^,^39^.

Figures 3b,d present the representative current (I) versus voltage (V) plots of solution-dropped and PDMS-transferred nanobeam devices, which were measured by varying the applied voltage within the range of 0 to 5 V and at constant temperature of 365 and 369.8 K, respectively (also see Supplementary Figures S7 and S8). The insulator-to-metal transition occurs at threshold voltages V_TH↑_ on the up-sweep and V_TH↓_ on the down-sweep. Similar to varying temperature, when altering voltage, the hysteresis width in voltage (ΔV_TH_) of the PDMS-transferred nanobeam device is larger than that of the solution-dropped nanobeam device. Also, both threshold voltages (V_TH↑_ and V_TH↓_) of the former device are higher than the latter device (see Table 1 and Supplementary Figure S6 for the statistical results). These trends could be originated from the tensile strain pre-existing in this PDMS-transferred nanobeam, which is consistent with the previous reports^17^,^28^,^29^. To further clarify the correlation between the strain and the hysteresis width, we also measured the temperature dependence of V_TH_ of VO_2_ nanobeams grown on a SiO_2_ layer (Supplementary Figure S9). The VO_2_ nanobeams are naturally pinned to the SiO_2_ layer and experience uniaxial tensile strain along c axis as a result of the growth in high temperature and thermal expansion mismatch between the nanobeam and the SiO_2_ layer^19^,^36^. Interestingly, the VO_2_ nanobeams grown on the SiO_2_ layer exhibit large widths in voltage hysteresis range, indicating that the amount of hysteresis can be correlated with the tensile strain in the VO_2_ nanobeams.

**Figure 4:**
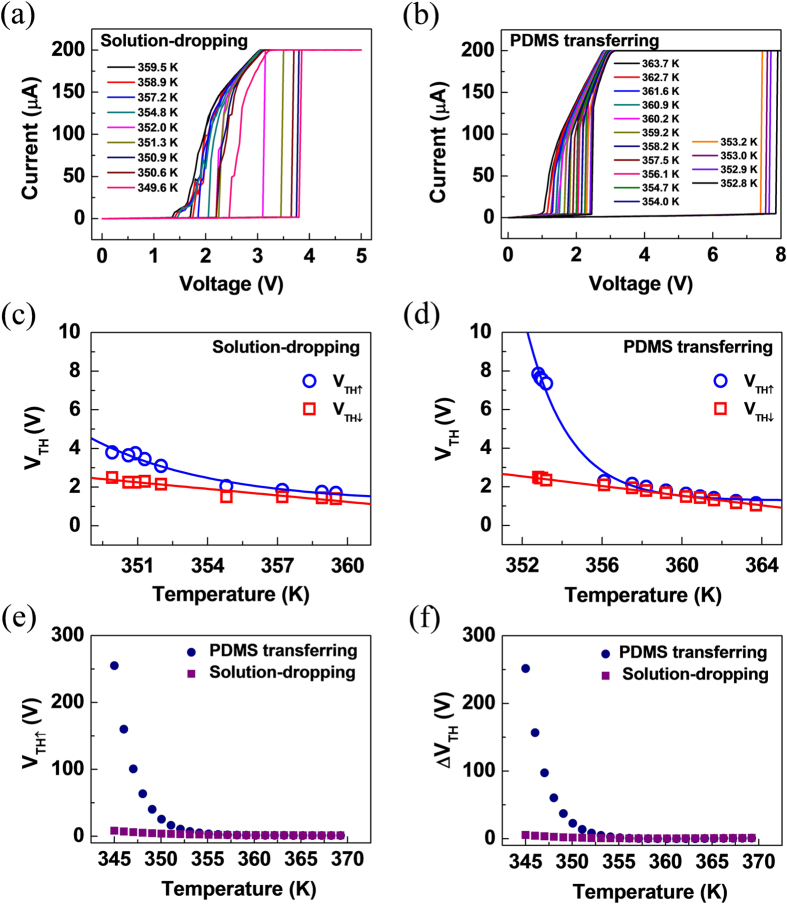
Relation between threshold voltages and temperatures in which the MIT occurs upon heating and cooling for a VO_2_ nanobeam. (**a**) Temperature-dependent I-V plots measured at compliance current (I_c_) = 200 μA and (**c**) temperature dependence of threshold voltage (V_TH_) for the two-terminal VO_2_ nanobeam device fabricated using a solution-dropping method. The corresponding plots for the PDMS-transferred nanobeam device are displayed in (**b**) and (**d**), respectively. Note that the blue data and fits are for the up-sweep from low to high bias voltages, and the red data and fits are for the down-sweep from high to low bias voltages. (e and f) V_TH↑_ vs. temperature and ΔV_TH_ (V_TH↑_ - V_TH↓_) vs. temperature plots of the solution-dropped and the PDMS-transferred nanobeams compared at the same temperature range. The plots are obtained from the fitted models.

Moreover, we understand the dependence of temperature on the threshold voltages (V_TH↑_ and V_TH↓_) of solution-dropped and PDMS-transferred nanobeam devices using the I-V plots at varied temperature, as shown in Fig. 4a,b, respectively. Note that we measured temperature-dependent I-V characteristics with a current compliance (I_c_) of 200 μA for two different types of VO_2_ nanobeam devices to reduce the joule heating caused from excessive current flow during the down-sweep from high to low bias voltages. Figures 4c,d present V_TH_ versus T plots of the corresponding nanobeam devices, demonstrating that V_TH↑_ exponentially increases with decreasing temperature, and these follow the relationship of V_TH↑_ ∝ exp(-T/T_0_). This indicates that the collective motion of carriers on the one-dimensional chain in charge-ordered systems, may play a dominant role for the insulator-to-metal transitions^2^,^46^. In contrast, V_TH↓_ is proportional to (T_MIT_ - T)^1/2^, implying that the Joule heating effect caused by scattering of charge carriers may be responsible for the metal-to-insulator transitions^2^,^47^. Apparently, the threshold voltages (V_TH↑_ and V_TH↓_) of both devices required to induce the MIT decrease with increasing temperature except for the case of V_TH↓_ in solution-dropped nanobeam device, indicating that the cooperative effect of thermal heating and electric field creates abrupt transitions for the tensile-strained VO_2_ nanobeam effectively. However, at high temperature range, the V_TH↑_ of the both devices remains nearly constant, representing the insulator-to-metal transition can be accomplished by a significant level of electric field though thermal heating is intensified. We note that the V_TH↓_ in solution-dropped nanobeam device is nearly constant with temperature, and its values are much smaller than the threshold voltages of the other cases at the similar range of temperature. We expect that these behaviors might be related to the fact that the strain resulted from the electrodes, and the adhesion between the nanobeam and the substrate is fully relieved at the voltages.

We further measured and analyzed the current-voltage characteristics for the nanobeam devices, especially with an upper I_c_ of 0.1 mA although it was difficult to define the threshold voltages for down-sweep from high to low bias voltages (V_TH↓_) (Supplementary Figure S10). Likewise, the dependence of temperature on V_TH↑_ and V_TH↓_ for the solution-dropped and PDMS-transferred nanobeam devices showed similar trends. Notably, the relationship of V_TH↑_ ∝ exp(-T/T_0_) for the PDMS-transferred nanobeam device exhibits a distinct feature compared with the solution-dropped nanobeam device. Figures 4e,f compare the V_TH↑_ and ΔV_TH_ (V_TH↑_ - V_TH↓_) of the two different types of nanobeam devices at the identical range of temperatures based on their parameters of the fitted models, respectively. Both V_TH↑_ and ΔV_TH_, in the PDMS-transferred nanobeam device provide larger values than those of the solution-dropped nanobeam device, confirming the presence of the tensile strain pre-existing in this PDMS-transferred nanobeam, as discussed in Fig. 2.

Since the conductivity in the insulating state is strongly dependent on temperature and the local temperature profile varies with bias, we considered the change in the conductance as a function of bias. Based on the previous literatures^36^,^48^,^49^,^50^, we carried out simulations by modeling local Joule heating using a COMSOL Multiphysics software in our system (Supplementary Figures S11). The parameters of VO_2_ nanobeams used in these simulations were 2.5 μm in length of the nanobeam, 100 nm in thickness of the nanobeam, and 220 nm in width of the nanobeam, electrical conductivity of 100 S/m, thermal conductivity of 6.5 W/m·K, and heat capacity of 690 J/kg·K. Note that for convenience of discussion and simplification of local Joule heating effects, we assumed a constant electrical conductivity at the temperature range of 300–340 K for the VO_2_ nanobeams. At these temperatures, the VO_2_ nanobeams showed completely insulating phases, as shown in Fig. 3, S7 and S8. As shown in Figure S11a, the temperature rise (colored symbols) was estimated against the applied voltage, and the results was matched well with the fitting curves calculated by 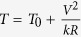, which are similar to the one used by Zimmers *et al.*^51^ Note that our VO_2_ nanobeam devices are different from the VO_2_ devices studied in Ref. 51 in which the VO_2_ channel is a thin film with a ribbon structure of 50 μm width, 10 and 20 μm channel length and the voltages applied across these VO_2_ channels vary with more than 40 voltages between electrodes. The equation expressed above was derived by a simple power dissipation model^49^. Here, T_0_ is ambient temperature, k is a collection of thermal parameters, including the thermal coefficient and heat capacity of the nanobeam and the underling substrate surface, V is the applied voltage, and R is the resistance of the VO_2_ nanobeam. When the applied bias voltage is as low as 5 V, the local Joule heating in the nanobeam could appear to be localized along the nanobeam. In this case, however, the joule heating is not sufficient to trigger the transition by itself without thermal heating when the electric field across the VO_2_ nanobeam, and a typical temperature rise is less than 10 K, consistent with a previous report^52^. In addition, the temperature profile estimated by COMSOL Multiphysics software (Supplementary Figure S11b) shows that while the applied bias voltage is as low as 2 V, the corresponding temperature along the nanobeam increases only several degrees enhancement, which is much smaller than temperature applied to the VO_2_ sample by a thermal heating stage. Therefore, we speculate that although the increase of applied bias voltage (or electric field) across the nanobeam can lower the potential barrier to carrier transport with increasing the carrier density, the electric field-induced local heating effect is not sufficient to trigger the insulator-metal transition in the nanobeam.

**Figure 5:**
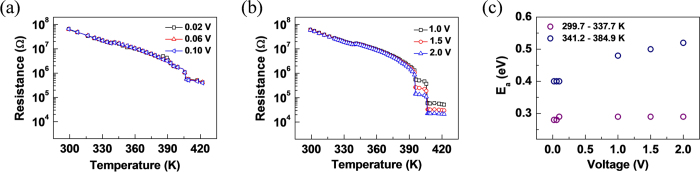
Resistance versus temperature plots. (**a**,**b**) Resistance *versus* temperature plots, measured by varying the applied bias in a range of (**a**) 0.02 – 0.1 V and (b) 1 – 2 V for the VO_2_ nanobeam devices fabricated by the PDMS- transferring method. (**c**) Thermal activation energies *versus* voltage plots of the insulating states occurring at 299.7 – 337.7 K (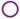) and at 341.2 – 384.9 K (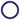) of Figures 5a,b).

Now, we focus on the role of applied voltage on the MIT for the two different types of nanobeam devices at varied temperature. Fig. 5a,b show the dependence of temperature on the insulator-to-metal transition of the PDMS-transferred nanobeam device by varying the applied voltage in the two different ranges. At the low voltages (V_DS_ = 0.02 – 0.1 V) (Fig. 5a), the resistance smoothly decreases with T and at ~406 K, it decreases discontinuously by only less than a half order. Note that the resistance changes almost independently with the applied voltage, and it only drops down to 411.7 kΩ. This result indicates that the almost entire portions of the nanobeams remain in insulating states, and a thermal heating effect alone is not sufficient to induce the abrupt transition to metallic phase as discussed above. On the other hand, when the applied voltages are high (V_DS_ = 1 – 2 V) (Fig. 5b), the abrupt steps in the resistance appear at 396 K and 406 K in the course of transforming into metallic phase. We suspect that the steps are attributed to the multiple nucleation of the rutile phases occurring randomly rather than an expansion of a single rutile phase while coexisting with insulating phases due to the inhomogeneous strain between the substrate and the nanobeam^2^,^19^,^41^. The total reduction in resistance is nearly two orders of magnitude with the insulator-to-metal phase transition completed at ~423 K. Interestingly, the activation energy in the range of temperature (341.2 – 384.9 K) increases significantly when the voltage above 1 V is applied whereas that in the range of temperature (299.7 – 337.7 K) remains constant, as presented in Fig. 5c. This result indicates that the increased voltage with the presence of heating helps the insulating phase such as M2 phase transform to the metallic rutile phase effectively.

## Conclusions

In conclusion, we have investigated the influence of substrate-mediated strain on the correlative role of thermal heating and electric field on the metal-insulator transitions of the tensile-strained VO_2_ nanobeams prepared by the PDMS-transferring method, compared with those of the nanobeams by the solution-dropping method. The plots of resistance versus temperature and current versus voltage for the insulator-to-metal transition and the metal-to-insulator transition showed that the hysteresis widths in temperatures and voltages of the phase transition were larger in the PDMS-transferred nanobeam. Moreover, the threshold voltages (V_TH_) needed to induce the insulator-to-metal transition and the metal-to-insulator transition decrease with increasing temperature, governed by a collective motion of carriers and a joule heating, respectively. Notably, these relationships for the PDMS-transferred VO_2_ nanobeam are distinct features compared with those of the solution-dropped nanobeam. Our study will provide helpful information on manipulating the properties of the VO_2_ nanobeam by altering temperature and voltage with a properly engineered strain.

## Additional Information

**How to cite this article**: Kim, M.-W. *et al.* Substrate-mediated strain effect on the role of thermal heating and electric field on metal-insulator transition in vanadium dioxide nanobeams. *Sci. Rep.* **5**, 10861; doi: 10.1038/srep10861 (2015).

## Supplementary Material

Supplementary Information

## Figures and Tables

**Table 1:** The parameters showing the MIT properties of the VO_2_ nanobeam devices fabricated using solution-dropping and PDMS-transferring methods.

**Mothods**	**ΔT_MIT_ (K)**	**T_MIT_ (K) upon heating**	**T_MIT_ (K) upon cooling**	**ΔV_TH_(V)**	**V_TH↑_ (V)**	**V_TH↓_ (V)**
Solution dropping	32.8±11.6	373.3±9.6	340.5±6.6	1.5±0.4	2.2±0.5	0.7±0.3
PDMS transferring	58.9±20.7	394.0±13.7	340±3.9	2.9±0.3	4.1±0.3	1.1±0.1

**
