# Supplementary material for: Substrate-mediated strain effect on the role of thermal heating and electric field on metal-insulator transition in vanadium dioxide nanobeams
Source: Sci Rep. 2015 Jun 4;5:10861. doi: 10.1038/srep10861 (PMC4455114; doi:10.1038/srep10861)
Supplement: Supplementary Information [file srep10861-s1.doc]

**SUPPLEMENTARY INFORMATION**

**Substrate-mediated strain effect on the role of thermal heating and electric field on metal-insulator transition in vanadium dioxide nanobeams**

Min-Woo Kim^1^, Wan-Gil Jung^1^, Hyun-Cho^1^, Tae-Sung Bae^2^, Sung-Jin Chang^3^, Ja-Soon Jang^4^, Woong-Ki Hong^2*^, Bong-Joong Kim^1*^

^1^School of Materials Science and Engineering, Gwangju Institute of Science and Technology, Gwangju 500-712, South Korea

^2^Jeonju Center, Korea Basic Science Institute, Jeonju, Jeollabuk-do 561-180, South Korea

^3^Department of Chemistry, Chung-Ang University, 84 Heukseok-ro, Dongjak-gu, Seoul 156-756, South Korea

^4^School of Electrical Engineering and Computer Science, Department of Electronics, Yeungnam University, Gyeongsangbuk-do 712-749, South Korea

^*^Corresponding Authors. E-mail address: (W.K.H) wkh27@kbsi.re.kr; (B.J.K) kimbj@gist.ac.kr

**Figure S1 TEM results of a single-crystalline VO_2_ nanobeam.** (a,b) Low-magnification bright field (BF) transmission electron microscopy (TEM) images of an as-grown VO_2_ nanobeam. (c) A high resolution TEM image of the VO_2_ nanobeam in marked by the blue box in Figure S1 (b). (d) The selective area electron diffraction (SAED) pattern of an as-grown VO_2_ nanobeam.

**Figure S2** **Statistical histograms of length and diameter for VO_2_ nanobeams.** Statistical histograms of (a) length and (b) diameter for VO_2_ nanobeams. (c) An optical image of VO_2_ nanobeams by a 100 × objective lens (numerical aperture = 0.9).

**Figure S3 Current (I)-voltage (V) characteristics and their corresponding resistance versus voltage plot.** Representative I-V characteristics measured at room temperature for two-terminal VO_2_ nanobeam devices fabricated by (a) solution-dropping and (c) PDMS-transferring methods. Resistance as a function of the applied voltage for (b) the solution-dropped VO_2_ nanobeam devices and (d) PDMS-transferred VO_2_ nanobeam devices.

**Figure S4 Temperature dependent resistance at V_DS_ = 0.02 V for the PDMS-transferred VO_2_ nanobeam.** Temperature dependence of the resistance during heating and cooling cycles (at V_DS_ = 0.02 V) for the VO_2_ nanobeam transferred by a PDMS-transferring method.

**Figure S5 Current (I)-voltage (V) characteristics at insulating and metallic states and their corresponding resistance-voltage plots.** Representative I-V characteristics measured at insulating and metallic states for (a,b) the solution-dropped VO_2_ nanobeam device and (c,d) the PDMS-transferred VO_2_ nanobeam device. Resistance as a function of the applied voltage for the insulating and metallic states of (e) the solution-dropped VO_2_ nanobeam device and (f) the PDMS-transferred VO_2_ nanobeam device.

**Figure S6** **Hysteresis widths in threshold voltages and temperatures for the solution-dropping and PDMS transfer VO_2_ nanobeam devices.** (a) Hysteresis widths in threshold voltages in which the MIT occurs upon heating and cooling for the solution-dropping and PDMS transfer VO_2_ nanobeam devices. (b) Hysteresis widths in temperatures in which the MIT occurs upon heating and cooling for the solution-dropping and PDMS transfer VO_2_ nanobeam devices.

**Figure S7 Representative I-V characteristics for a solution-dropped VO_2_ nanobeam.** I-V characteristics measured at (a) 361 K, (b) 362 K, (c) 363 K, (d) 364 K, (e) 365 K, and (f) 366 K under compliance current (I_c_) = 0.1 mA.

**Figure S8 Representative I-V characteristics for the transition temperature of a PDMS-transferred VO_2_ nanobeam.** I-V characteristics measured at (a) 368.1 K, (b) 369.8 K, (c) 370.5 K, (d) 371.6 K, (e) 376.1 K, and (f) 377.5 K under compliance current (I_c_) of 0.1 mA.

**Figure S9 Representative current (I)-voltage (V) characteristics for VO_2_ nanobeams grown on a SiO_2_ layer.** Representative I-V characteristics measured at (a) T = 356 K and (b) T= 361 K by varying the applied voltage both in the forward- and reverse-sweep for VO_2_ nanobeams grown on a SiO_2_ layer.

**Figure S10 Temperature-dependent current (I)-voltage (V) characteristics and the relation between V_th_ and temperature.** (a) Representative Temperature-dependent I-V characteristics measured at compliance current (I_C_) of 0.1 mA for the two-terminal VO_2_ nanobeam device fabricated using a solution-dropping method. (b) I-V characteristics measured at I_C_ = 0.1 mA for the two-terminal VO_2_ nanobeam device fabricated using a PDMS-transferring method. (c) Temperature dependence of threshold voltage (V_TH_) for the two-terminal VO_2_ nanobeam device fabricated using a solution-dropping method. (d) Temperature dependence of V_TH_ for the two-terminal VO_2_ nanobeam device fabricated using a PDMS-transferring method.

**Figure S11 Simulation results showing temperature changes against the applied voltage.** (a) Temperature changes against the applied voltage. The fitting curves are in good agreement with the simulation data. (b) Simulated temperature variation at T = 328 K and V = 2 V in VO_2_ nanobeam on the SiO_2_/Si substrate.
